# Relationship between trace elements status and atrial fibrillation in patients with valvular heart diseases

**DOI:** 10.3389/fcvm.2025.1691845

**Published:** 2025-11-07

**Authors:** Xiang Liu, Yijia Shao, Zirui Huang, Jiayin Huang, Linjiang Han, Shaoyou Lu, Haijiang Guo, Jian Liu

**Affiliations:** 1 Department of Cardiac Surgery, Guangdong Cardiovascular Institute, Guangdong Provincial People’s Hospital (Guangdong Academy of Medical Sciences), Southern Medical University, Guangzhou, China; 2 Guangdong Provincial Key Laboratory of South China Structural Heart Disease, Guangzhou, China; 3 Department of Geriatrics, The First Affiliated Hospital, Sun Yat-sen University, Guangzhou, China; 4 School of Public Health (Shenzhen), Sun Yat-sen University, Shenzhen, China

**Keywords:** atrial fibrillation, valvular heart disease, trace element, exposure, risk factor

## Abstract

**Background:**

Atrial fibrillation (AF) combined with valvular heart disease (VHD) is increasingly prevalent worldwide and is associated with high morbidity and mortality. Studies have showed trace element concentrations varied in patients with AF and may be involved in the pathogenesis of AF. However, no data is currently available for those with VHD.

**Methods:**

Urine samples as well as clinical data were collected from 72 VHD patients with AF (VHDAF) and 72 VHD patients without AF (VHD) and further analyzed for a panel of trace elements: lithium, manganese, cobalt, copper, zinc, arsenic, selenium, strontium, Cadmium, mercury, thallium, lead. Quantile g-computation was adopted to explore the joint effect of the 12 trace elements on AF in this population, and internal validation was performed using 1,000 bootstrap re-samples.

**Results:**

Compared with the VHD group, Sr levels were reduced, while Mn, Cu and Hg concentrations were increased in the VHDAF group. Quantile g-computation model indicated a significant association between the mixtures of the 12 urinary trace elements and AF in patients with heart valve disease (adjusted OR: 2.051; 95% CI: 1.180–3.565; *p* = 0.011). Positive partial effect was owing to Cu (weight: 0.43) and Hg (weight: 0.18), while negative partial effect mainly attributed to Sr (weight: 0.43) and Li (weight: 0.23).

**Conclusion:**

VHDAF patients had higher Cu levels and lower Sr levels, and the two elements have been supposed to exert the largest influence on AF. Further research is needed to establish the causal relationships.

## Introduction

Atrial fibrillation (AF) is a highly prevalent arrhythmia (1), and estimated to affect more than 30 million people worldwide (2). Valvular heart disease (VHD), which primarily include calcific aortic valve disease and degenerative mitral valve disease, is a significant contributor to cardiovascular morbidity and mortality (3, 4). On one hand, AF and VHD share common etiologies, including, but not limited to diabetes, obesity, aging, hyperlipidemia, and hypertension (4). On the other hand, there are complex interactions between the two diseases (4–6). Consequently, the prevalence of AF in VHD is very high, especially among those with moderate to severe valve disease (4).

Trace elements, including copper (Cu), manganese (Mn), iron (Fe), zinc (Zn), cobalt (Co) and non-metal selenium (Se), are crucial for normal growth, development, and physiological functions (7, 8). Of the human body, 3% is trace elements (9). Epidemiological evidence suggests that trace elements are strongly linked to cardiovascular health, with associations found in conditions such as VHD (10, 11) and acute myocardial infarction (12). And the results of animal experiments have also shown that exposure to trace elements can cause serious damages to the cardiovascular system (13, 14). Furthermore, recent studies showed that trace element concentrations varied in patients with AF and may be involved in the pathogenesis of AF (2, 15). However, no data is currently available for those with VHD.

In this study, the urinary concentrations of 12 trace elements were investigated from 72 VHD patients with AF (VHDAF) and 72 VHD patients without AF (VHD). And Quantile g-computation was adopted to explore the joint effect of the 12 trace elements on AF in this population, and internal validation was performed using 1,000 bootstrap re-samples.

## Methods

### Study population and sample collection

This population-based case-control study was conducted on 72 VHD patients with AF and 72 VHD patients without AF in Guangdong Provincial People's Hospital between December 2022 and May 2023. All patients were diagnosed with VHD by medical history, physical examination, laboratory examination, electrocardiography and echocardiography, and underwent scheduled surgery according to clinical guidelines. Coronary artery disease was excluded by coronary computed tomography angiography and/or coronary angiography. Participants with congenital heart disease, coronary heart disease which require clinical intervention, infective endocarditis, infectious diseases, severe traumas, or having surgeries within one month before sampling were excluded. The first-morning spot urine sample (10 mL) was obtained from all participants after they were diagnosed by VHD, with or without AF. These specimens were collected into a sterile polypropylene centrifuge tube (CorningScience, Mexico), and transported to the laboratory and placed in a −20℃ refrigerator within 2 h of collection and then transferred to −80℃ within 1 day. Demographic and clinical characteristics, including age, sex, tobacco smoking, heart rate, blood pressure, fasting blood glucose (FBG), serum creatinine (Scr), uric acid (UA), calcium (Ca), phosphorus (P), sodium (Na), potassium (K), alanine aminotransferase (ALT), aspartate aminotransferase (AST), serum total cholesterol (TC), triglyceride (TG), high-density lipoprotein cholesterol (HDL-C), low-density lipoprotein cholesterol (LDL-C), ejection fraction (EF), aortic cross-clamping (ACC) time, cardiopulmonary bypass (CPB) time, Operation time, ventilatory support (VS) time, intensive care unit (ICU) stay, hospital stay and complications were collected from the electronic medical records. All the patients received diuretics and digoxin before surgery. If patients had been taking anti-hypertension, diabetes, or dyslipidemia treatment medication, they were to continue using these drugs. For patients at risk of thrombosis, such as those with mitral stenosis combined with AF, low-molecular-weight heparin was administered for preoperative thromboembolism prophylaxis. This study was approved by the Ethics Committee of Guangdong Provincial People's Hospital (KY-Q-2021-062-04) and signed informed consent was obtained from all patients.

### Reagent and materials

Multi-element standard solution containing lithium (Li), Mn, Co, Cu, Zn, arsenic (As), strontium (Sr), Cadmium (Cd), thallium (Tl), and lead (Pb), single-element standard solutions of Se and mercury (Hg), and internal standards containing bismuth (Bi), germanium (Ge), indium (In), rhodium (Rh), scandium (Sc), terbium (Tb), and yttrium (Y) were purchased from Agilent Technologies (Santa Clara, CA, USA). Extra pure 68% nitric acid was obtained from Merck (Darmstadt, Germany). Deionized water with a resistivity of 18 MΩ cm was obtained from Millipore Purification Systems (Billerica, MA, USA).

### Sample preparation and analysis

Prior to analysis, all urine samples were thawed at 4℃, mixed, and centrifuged (8,000 rpm for 5 min). And 0.5 mL of the supernatant was transferred into a 15 mL polyethylene centrifuge tube, subsequently diluted 20-fold by adding 9.5 mL of 1% nitric acid solution and thoroughly mixed. For the blank, the urine sample was replaced by deionized water. Finally, an inductively coupled plasma mass spectrometer (ICP-MS) (Agilent Technologies, Santa Clara, CA, USA) was used to analyze the urinary concentrations of the metals (Li, Mn, Co, Cu, Zn, As, Sr, Cd, Hg, Tl, Pb) and non-metal Se using an kinetic energy discrimination (KED) mode. All the sample analysis protocols were in accordance with the Chinese national standard GBZ/T 308–2018. The instrumental parameters are presented in Supplementary Table S1.

### Quality assurance and quality control

A blank sample was prepared for each batch of 12 samples to assess background contamination, with no analytes detected. The standard curves for the 12 trace elements ranged from 0 to 200 μg/L, with correlation coefficients (R²) exceeding 0.999 for all elements (Supplementary Table S2). The limits of detection (LOD) were defined as three times the background signal of the matrix blank and the LOD for Li, Mn, Co, Cu, Zn, As, Se, Sr, Cd, Hg, Tl, and Pb were 0.034, 0.006, 0.002, 0.465, 0.465, 0.051, 0.527, 0.048, 0.006, 0.003, 0.006, and 0.008 μg/L, respectively. Concentrations below the LOD were assigned a value of LOD/√2. In order to evaluate the precision of analysis, the artificial urine samples were spiked with three different concentrations (1, 2, and 5 μg/L) of a standard metal solution to calculate recovery rates. The spiked recoveries ranged from 81.4% to 116.8%, with relative standard deviations less than 10%. Glass materials were avoided throughout the procedure to minimize background contamination.

### Statistical analysis

SPSS version 20.0 (SPSS, Chicago, IL) and R software version 4.5.1 (R Foundation for Statistical Computing) were used for statistical analysis. The Kolmogorov Smirnov test was used to test for normal distribution. Normally distributed variables are presented as mean ± standard deviation, and independent samples *t*-Test was used to compare means between independent samples. Non-normally distributed variables were expressed as median (interquartile range), and the Mann Whitney *U*-Test was employed for statistical comparisons. Categorical variables are presented as frequencies and Pearson's chi-square test was used for comparison. Correlation plots were drawn using the corrplot function from the corrplot package (version 0.95) in R. Statistical analysis of the 12 trace elements between the two groups and plots were performed in R software using RColorBrewer (version 1.1-3), ggpubr (version 0.6.1) and ggplot2 (version 3.5.2) packages. Quantile g-computation regression was conducted using the qgcomp package (version 2.18.4) with R studio, and the reliability of the results were estimated using a bootstrap analysis of 1,000 replicates. Because of the differences existing between the two groups, we used Na, ALT, AST and EF as covariates in the analyses. A two-tailed *p* value of less than 0.05 was defined as statistically significance.

## Results

### Demographic and clinical information of the study patients

The demographic and clinical information can be found in Table 1. There were 72 patients in the VHD group with an median age of 56.0 years (with an IQR of 48.0–59.0) and 72 patients in the VHDAF group with an median age of 54.0 years (with an IQR of 48.8–59.0). Compared to the reference group, VHDAF group had lower Na, higher ALT and AST. Notably, AF patients have lower left ventricular EF, longer mechanical ventilation time and ICU stay. Postoperative complications consisted of infection, arrhythmia, cardiac insufficiency, bleeding and electrolyte disturbances. No significant difference in the overall complication was detected between the VHD and VHDAF groups, and none died during hospitalization.

**Table 1:** Demographic information and clinical measurements of the reference group and VHDAF patients.

	VHD group (*n* = 72)	VHDAF patients (*n* = 72)	*p* value
Age, y	56.0 (48.0, 59.0)	54.0 (48.8, 59.0)	0.986
Sex, female (%)	34 (47.2)	34 (47.2)	1.000
BMI	23.8 ± 3.7	23.2 ± 3.8	0.331
Smoking (%)	13 (18.1)	11 (15.3)	0.655
HR, bpm	78.7 ± 11.7	80.7 ± 13.0	0.331
SBP, mmHg	125.3 ± 20.7	121.7 ± 14.6	0.224
DBP, mmHg	74.3 ± 13.3	74.9 ± 12.2	0.774
FBG, mmol/L	5.0 (4.6, 5.4)	5.1 (4.7, 5.5)	0.681
Scr, μmol/L	78.3 (61.5, 94.9)	78.5 (66.1, 92.7)	0.803
UA, μmol/L	408.3 ± 129.5	409.4 ± 122.3	0.959
Ca, mmol/L	2.3 (2.3, 2.4)	2.3 (2.2, 2.4)	0.334
P, mmol/L	1.3 (1.2, 1.4)	1.2 (1.1, 1.4)	0.376
Na, mmol/L	139.9 (138.5, 142.0)	139.3 (137.4, 140.9)	0.022
K, mmol/L	4.1 (3.9, 4.2)	4.0 (3.8, 4.3)	0.701
ALT, U/L	18 (13, 29)	25 (16, 33)	0.005
AST, U/L	22 (17, 37)	34 (24, 52)	0.001
TC, mmol/L	4.9 (4.5, 5.7)	4.8 (4.1, 5.5)	0.117
TG, mmol/L	1.3 (1.1, 1.5)	1.2 (0.9, 1.7)	0.584
HDL, mmol/L	1.3 (1.1, 1.5)	1.3 (1.1, 1.4)	0.546
LDL, mmol/L	3.0 (2.7, 3.4)	2.9 (2.3, 3.4)	0.203
EF, %	64 (62, 67)	61 (58, 65)	0.007
ACC time, min	91 (64, 124)	85 (64, 126)	0.924
CPB Time, min	145 (110, 189)	156 (119, 198)	0.228
Operation time, min	250 (208, 306)	257 (219, 318)	0.263
VS time, h	12.0 (6.4, 20.3)	16.2 (9.4, 21.0)	0.044
ICU stay, d	1.6 (0.9, 2.6)	1.8 (1.5, 2.8)	0.023
Hospital stay, d	11.0 (12.0, 19.8)	15.0 (13.0, 21.5)	0.233
Complications (%)			0.171
Infection	6 (8.3)	8 (11.1)	
Arrhythmia	13 (18.1)	5 (6.9)	
Cardiac insufficiency	2 (2.8)	5 (6.9)	
Other	3 (4.2)	2 (2.8)	

BMI, body mass index; HR, heart rate; SBP, systolic blood pressure; DBP, diastolic blood pressure; FBG, fasting blood glucose; Scr, serum creatinine; UA, uric acid; Ca, calcium; P, phosphorus; Na, sodium; K, potassium; ALT, alanine aminotransferase; AST, aspartate aminotransferase; TC, total cholesterol; TG, triglyceride; HDL, high density lipoprotein; LDL, low density lipoprotein; EF, ejection fraction; ACC, aortic cross-clamping; CPB, cardiopulmonary bypass; VS, ventilatory support; ICU, intensive care unit.

### Spearman correlations between urinary trace elements

**Figure 1:**
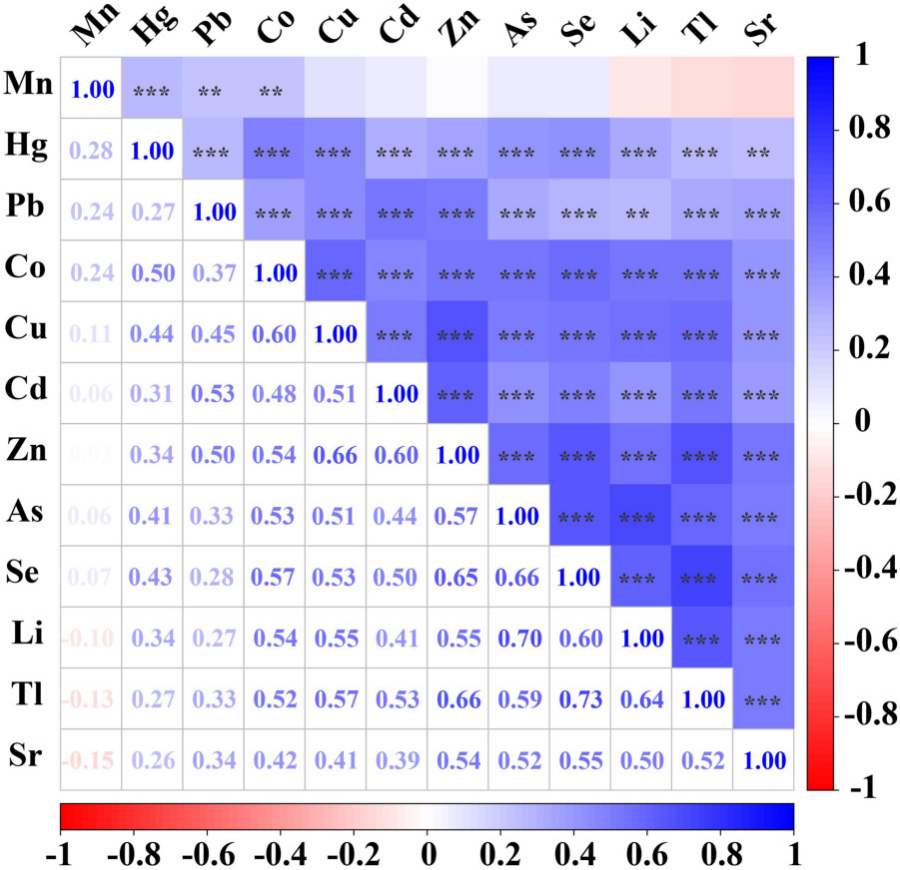
Spearman correlations between urinary trace elements. Correlations were observed between most of the trace elements examined. Mn concentrations were significantly correlated with Hg, Pb and Co. Hg concentrations were significantly correlated with Pb, Co, Cu, Cd, Zn, As, Se, Li, Tl and Sr. Pb concentrations were significantly correlated with Co, Cu, Cd, Zn, As, Se, Li, Tl and Sr. Co concentrations were significantly correlated with Cu, Cd, Zn, As, Se, Li, Tl and Sr, with correlation coefficients equal to or exceeding 0.6 for Cu. Cu concentrations were significantly correlated with Cd, Zn, As, Se, Li, Tl and Sr, with correlation coefficients equal to or exceeding 0.6 for Zn. Cd concentrations were significantly correlated with Zn, As, Se, Li, Tl and Sr, with correlation coefficients equal to or exceeding 0.6 for Zn. Zn concentrations were significantly correlated with As, Se, Li, Tl and Sr, with correlation coefficients equal to or exceeding 0.6 for Se and Tl. As concentrations were significantly correlated with Se, Li, Tl and Sr, with correlation coefficients equal to or exceeding 0.6 for Se and Li. Se concentrations were significantly correlated with Li, Tl and Sr, with correlation coefficients equal to or exceeding 0.6 for Li and Tl. Li concentrations were significantly correlated with Tl and Sr, with correlation coefficients equal to or exceeding 0.6 for Tl. Tl concentrations were significantly correlated with Sr. The lower left panel shows Spearman's rank correlation coefficients, and correlations were considered strong when the correlation coefficient exceeded 0.60. The top right panel shows their significance (***p < 0.001, **p < 0.01).

Pairwise correlations between urinary trace elements are shown in Figure 1. Mn concentrations were significantly correlated with Hg, Pb and Co. Hg concentrations were significantly correlated with Pb, Co, Cu, Cd, Zn, As, Se, Li, Tl and Sr. Pb concentrations were significantly correlated with Co, Cu, Cd, Zn, As, Se, Li, Tl and Sr. Co concentrations were significantly correlated with Cu, Cd, Zn, As, Se, Li, Tl and Sr, with correlation coefficients equal to or exceeding 0.6 for Cu. Cu concentrations were significantly correlated with Cd, Zn, As, Se, Li, Tl and Sr, with correlation coefficients equal to or exceeding 0.6 for Zn. Cd concentrations were significantly correlated with Zn, As, Se, Li, Tl and Sr, with correlation coefficients equal to or exceeding 0.6 for Zn. Zn concentrations were significantly correlated with As, Se, Li, Tl and Sr, with correlation coefficients equal to or exceeding 0.6 for Se and Tl. As concentrations were significantly correlated with Se, Li, Tl and Sr, with correlation coefficients equal to or exceeding 0.6 for Se and Li. Se concentrations were significantly correlated with Li, Tl and Sr, with correlation coefficients equal to or exceeding 0.6 for Li and Tl. Li concentrations were significantly correlated with Tl and Sr, with correlation coefficients equal to or exceeding 0.6 for Tl. Tl concentrations were significantly correlated with Sr.

### Boxplot analysis of the urinary trace elements between VHD and VHDAF group

**Figure 2:**
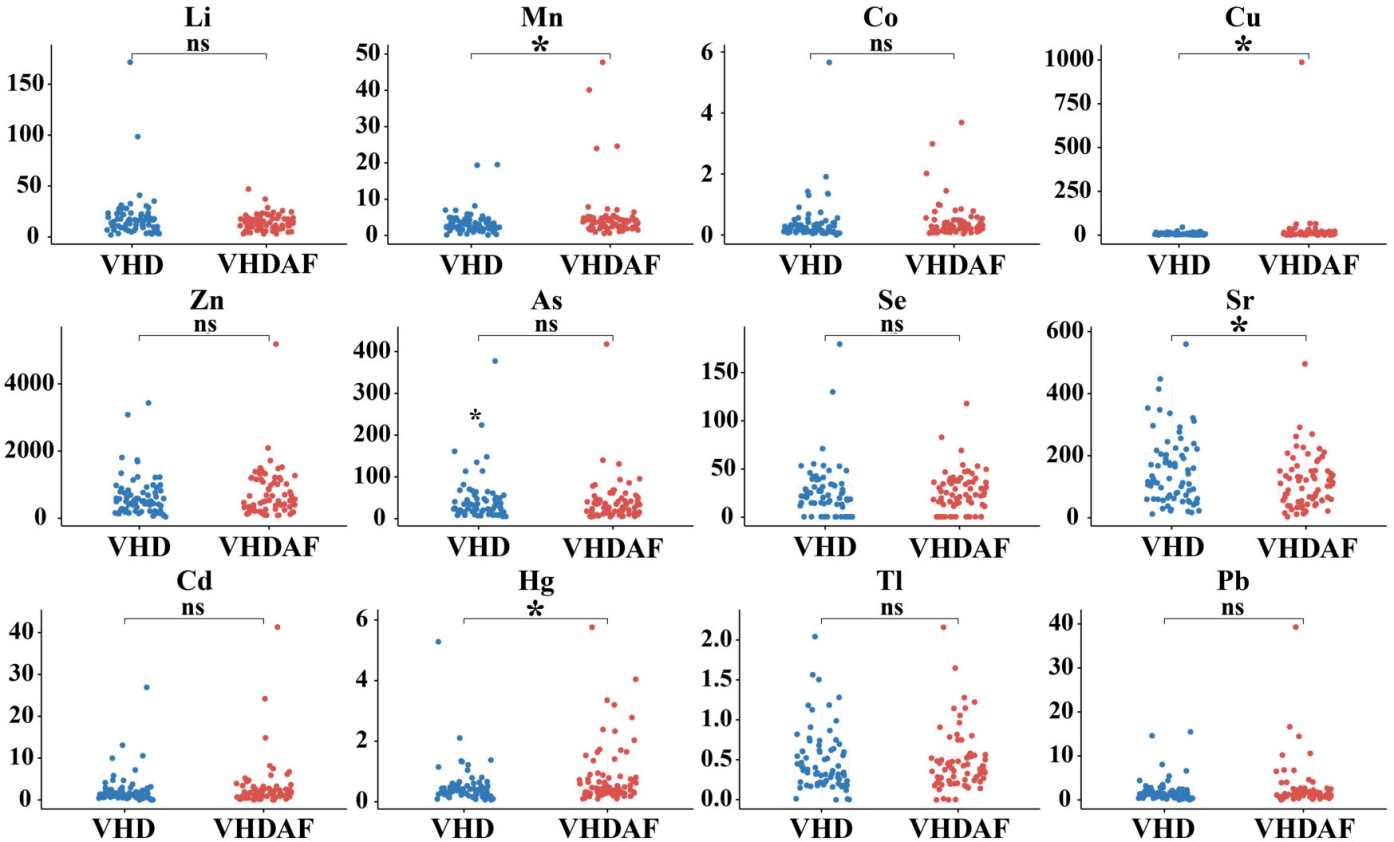
Boxplot analysis of the urinary trace elements between VHD and VHDAF group. Compared with the VHD groups, Mn, Cu and Hg levels were increased, and Sr levels were decreased in the VHDAF group. The blue Boxplot represent VHD group, red Boxplot represent VHDAF group. *p < 0.05 vs. the VHD group.

As shown in Figure 2, Mn, Cu, Sr and Hg were found to be significantly different between the two groups. Specifically, Mn, Cu and Hg levels were increased, and Sr levels were decreased in the VHDAF group compared with the control groups. There were no significant differences in the concentration of Li, Co, Zn, As, Se, Cd, Tl and Pb between the two groups. Detailed results are also shown in Table 2.

**Table 2:** Urinary concentrations of metals from VHDAF patients and the reference group.

	VHD group (*n* = 72)	VHDAF group (*n* = 72)	*p* value
Li, ppb	14.81 (8.72, 21.26)	14.13 (9.29, 18.34)	0.496
Mn, ppb	2.69 (1.72, 4.19)	3.61 (2.21, 4.63)	0.029
Co, ppb	0.22 (0.10, 0.37)	0.25 (0.16, 0.44)	0.201
Cu, ppb	7.13 (4.23, 10.38)	9.20 (6.76, 14.81)	0.021
Zn, ppb	506.51 (232.28, 794.90)	518.56 (266.59, 1,148.17)	0.390
As, ppb	25.30 (15.09, 47.89)	28.40 (13.98, 47.00)	0.868
Se, ppb	26.98 (18.62, 39.26)	29.74 (19.70, 39.58)	0.693
Sr, ppb	120.19 (60.87, 207.79)	101.77 (53.39, 150.63)	0.044
Cd, ppb	1.40 (0.88, 2.36)	1.66 (0.92, 3.10)	0.432
Hg, ppb	0.37 (0.24, 0.525)	0.47 (0.28, 0.85)	0.034
Tl, ppb	0.40 (0.26, 0.65)	0.42 (0.28, 0.56)	0.917
Pb, ppb	1.31 (0.78, 2.32)	1.36 (0.76, 2.23)	0.609

ppb, parts per billion; Li, lithium; Mn, manganese; Co, cobalt; Cu, copper; Zn, zinc; As, arsenic; Se, selenium; Sr, strontium; Cd, Cadmium; Hg, mercury; TI, thallium; Pb, lead.

### Weights of each trace element by quantile g-computation regression for AF

**Figure 3:**
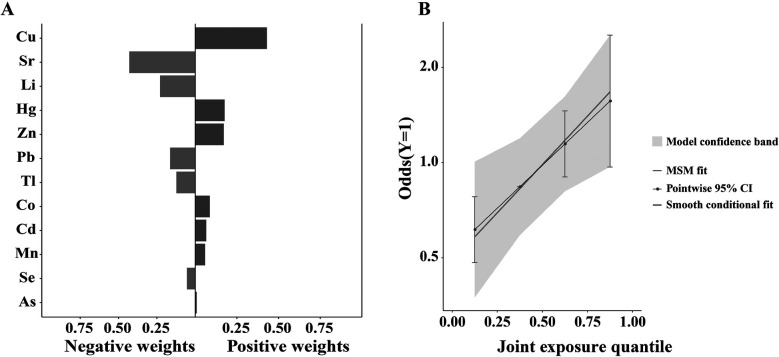
Weights of each trace element by quantile g-computation regression for AF. **(A)** In the quantile g-computation model, the weights representing the proportion of the positive or negative partial effect for each trace element for AF. Positive partial effect was owing to Cu (weight: 0.43) and Hg (weight: 0.18), while negative partial effect mainly attributed to Sr (weight: 0.43) and Li (weight: 0.23). **(B)** Further pooled risk estimates for AF suggest an increasing trend toward higher AF risk with increasing exposure to these trace elements mixtures. After adjusting for covariates including Na, ALT, AST and EF, there was statistically significant association between the mixtures of the 12 urinary trace elements and AF in patients with heart valve disease (adjusted OR: 2.051; 95% CI: 1.180–3.565; *p* = 0.011).

In the quantile g-computation model, the weights representing the proportion of the positive or negative partial effect for each trace element for AF, are shown in Figure 3A. Positive partial effect was owing to Cu (weight: 0.43) and Hg (weight: 0.18), while negative partial effect mainly attributed to Sr (weight: 0.43) and Li (weight: 0.23). Further pooled risk estimates for AF suggest an increasing trend toward higher AF risk with increasing exposure to these trace elements mixtures, as presented in Figure 3B. There was statistically significant association between the mixtures of the 12 urinary trace elements and AF in patients with HVD (unadjusted OR: 1.725; 95% CI: 1.086–2.741; *p* = 0.021). After adjusting for covariates including Na, ALT, AST and EF, this statistical difference still existed (adjusted OR: 2.051; 95% CI: 1.180–3.565; *p* = 0.011). These results indicate that exposure to these elements mixtures confers to an increased risk of AF in this population.

## Discussion

In this research, most of the OH-PAH pairs exhibited moderate to strong positive correlations, suggesting that these OH-PAHs in the population likely originated from the same or similar contamination sources. We found that Sr levels were reduced, while Mn, Cu and Hg concentrations were increased in the VHDAF group. Furthermore, quantile g-computation models identified a significant association with the mixtures of the 12 urinary trace elements and AF in patients with VHD. Cu was assigned the largest weight for the partial positive effect in the mixtures, and Sr was assigned the largest weight for the partial negative effect in the mixtures.

Previous research has indicated disproportions in the essential/toxic metals in the blood may be involved in pathogenesis of the VHD (16). It was found that, compared to those of the healthy subjects, VHD patients are characterized by significantly higher levels of Cu, Fe, Mg, Mn and Sr in the scalp hair (17). A systematic review and meta-analysis found a significant association between Cu and cardiovascular disease (CVD) (18). While Cu and Zn deficiency may also be associated with a higher risk of CVD such as valvular regurgitation (10). According to a recent study by Sun et al., Cu is positively correlated with the prevalence and severity of acute myocardial infarction (12). There have been reported excess of Cu is toxic to living cells. Cu overload can cause cuproptosis by direct binding to lipoylation proteins in the tricarboxylic acid cycle, leading to lipoylated protein aggregation and reducing iron-sulfur cluster protein levels, thereby leading to proteotoxic stress and cell death. Accumulating evidence suggests cuproptosis plays an essential role in the development and pathogenesis of CVD (19). Serum Cu levels were found to be positively correlated with lipoprotein(a), an inflammatory marker, in patients with aortic stenosis (11). As for Hg, it has a strong affinity for sulfhydryl groups on molecules such as glutathione, cysteine, metallothionein, and other sulfur-containing antioxidants in the body, which affects anti-oxidant defense systems, ultimately resulting in mitochondrial dysfunction and subsequent mitochondrial reactive oxygen species (ROS) accumulation and increased oxidative stress. Clinical consequences of Hg toxicity include atherosclerosis, coronary heart disease, hypertension, arrhythmias and renal dysfunction (20). The same results have been found in animal experiments. In an experimental study examining the effects of Cu, Mn, and Hg on blood parameters, as well as the aorta and heart in rats, it was found that exposure to these metals led to disruption in the arrangement of aortic collagen and elastin bands, as well as the structural integrity of cardiac mitochondria and myofibrils (14). Mice on a high-Mn diet exhibit changes in Mn levels and distribution within infected tissues, leading to increased virulence of S. aureus and heightened heart infection. Mechanistically, S. aureus utilizes bioavailable Mn to neutralize reactive oxygen species and protect itself against neutrophil attacks, thereby enhancing its survival in the heart (13).

However, for Sr, the evidences of it's potential effects in CVD are still limited and conflicting. Sr was found to be positively associated with the risk of incident CVD in patients with type 2 diabetes (21). Preeclampsia is characterized by systemic vascular endothelial dysfunction, leading to the hypertension and proteinuria. Sr levels and oxidative status were significantly higher in preeclamptic women than in control groups (22, 23). Yet, another study reported that, although the serum concentrations of Sr were significantly increased in smokers compared with nonsmokers, its effect on endothelial cell transcription was not significant (24). Conversely, it was found that Sr may have beneficial effects on the cardiovascular system. Wang et al. (25) showed each unit increase in urinary Sr is associated with a 1.1% decrease in the risk for CVD. Besides, Sr has been linked to the promotion of angiogenesis. Sr has been reported to be able to promote angiogenesis by increasing the expressions of pro-angiogenic factors (vascular endothelial growth factor and basic fibroblast growth factor) (26). Furthermore, *ex vivo* and *in vivo* assays confirmed that Sr can protect hearts against myocardial ischemia/reperfusion injury by reducing cardiomyocyte apoptosis and promoting angiogenesis (27).

By comparison, the relationship between heavy metal exposure and arrhythmic events is rarely reported. Previous studies indicate that Pb, Cd and Hg can interfere with calcium transport systems such as calcium channels and calcium pumps and thereby disturbing calcium homeostasis (28, 29). Calcium homeostasis is essential to normal electrical activity of the heart and disrupted calcium homeostasis can lead to arrhythmia (30, 31). Cardiac repolarization and arrhythmias caused by long QT wave are significantly influenced by potassium channel which is encoded by the human ether-a-go-go related gene (hERG) (32). Long-term exposure to arsenic trioxide can increase cardiac calcium currents and reduce hERG expression and ultimately lead to prolongation of the QT interval (33). In the present study, we found that VHDAF patients had higher Cu levels and lower Sr levels, and moreover, the two elements have been supposed to exert the largest influence on AF. Clinically, high serum Cu levels have been shown to be associated with arrhythmias. It may be due to the Cu-based enzymes entering the serum after myocardium damage, or sympathetic nerves triggering the release of Cu into the blood from liver stores during stress (19). Experiments showed that bradycardia and heartbeat irregularity can be observed in zebrafish exposed to Cu and these effects can be rescued by the glycyl-histidyl-lysine tripeptides (34). Wilson's disease, an autosomal recessive disorder related to Cu accumulation, resulting from mutations in the Wilson disease protein (35). Cu accumulation within the myocardium can induce ventricular fibrillation and tachycardia in these patients, which is the most frequent cardiac presentation of Wilson's disease (36). And neosulpirin, as chelating agent for Cu, could be effective in preventing the formation of ROS and arrhythmias after myocardial ischemia/reperfusion injury (37). However, no evidence has been reported regarding the association between Sr and arrhythmias. AF and chronic coronary syndrome share a strong bidirectional relationship. Chronic coronary syndrome alters the structure and function of gap junction proteins, impairing action potential conduction and causing ischemic necrosis of cardiomyocytes, which are subsequently replaced by fibrous tissue. This remodeling promotes the maintenance of focal ectopic activity in the atrial myocardium (38). Sr has previously been reported to promote angiogenesis and ameliorate myocardial ischemia/reperfusion injury (26, 27), which suggests that Sr may exert its antiarrhythmic effect by suppressing myocardial apoptosis and improving myocardial remodeling. According to another study, acute atrial ischemia is a well-established contributor to postoperative AF, and insulin can reduce the risk of ischemia/reperfusion-induced AF by improving the electrophysiological properties and calcium handling of atrial cardiomyocytes (39). The evidence presented here is correlational, and therefore, further research is needed to clarify causal relationships between these trace elements and AF risk in VHD patients.

There were several limitations in this research. First, a formal sample size calculation was not conducted prior to data collection. Instead, the sample size was determined based on practical considerations and our previous study (40). In addition, the sample size is limited, and future research with a larger sample size is needed to further validate these findings. Second, we did not gather data on potential sources of trace element exposure, such as dietary habits, occupational exposure, environmental factors, or supplement use. Moving forward, we plan to collect more detailed data on dietary intake and environmental exposure to trace elements, which could offer valuable insights for clinical and public health recommendations. Third, although the postoperative complication rates in both patient groups were similar and appeared to be influenced by the poor condition of these patients and the technical performance of the surgery itself, we cannot exclude the possibility that some of these complications may be linked to AF and trace element levels.

## Conclusion

In this study, we found that Sr levels were reduced, while Mn, Cu and Hg concentrations were increased in patients with AF and significant valvular lesions. Among them, Cu is suggested to be the most important contributor to AF, and conversely, Sr is considered the most important mediator of the beneficial effect for AF. However, this study was only a preliminary correlation analysis between the trace elements and AF risk in VHD patients, and further research is needed to establish the causal relationships.

## Data Availability

The original contributions presented in the study are included in the article/Supplementary Material, further inquiries can be directed to the corresponding authors.
