# Supplementary material for: Relationship between trace elements status and atrial fibrillation in patients with valvular heart diseases
Source: Front Cardiovasc Med. 2025 Nov 7;12:1691845. doi: 10.3389/fcvm.2025.1691845 (PMC12634502; doi:10.3389/fcvm.2025.1691845)
Supplement: Supplementary file 1 [file Table1.docx]

**Table S1**. Instrumental parameters of the inductively coupled plasma mass spectrometer

| Parameters | Values |
| --- | --- |
| RF forward power | 1500 W |
| Cooling air flow rate | 15 L/min |
| Carrier gas flow rate | 1 L/min |
| Auxiliary gas flow rate | 1 L/min |
| Scanning mode | Full quantification |
| Integration time | 0.1 s |
| Times of acquiring data | 3 |
| Mode | Collision mode |
| Cell entrance | -40 V |
| Cell exit | -38 V |
| Octopole bias | -16 V |
| Quadrupole bias | -13 V |
| Online internal standard (concentration) | 10.0 μg/mL |

**Table S2**. Standard curves and correlation coefficients (R^2^)

| Metals | Standard curves | Correlation coefficients (R^2^) |
| --- | --- | --- |
| Li | y=0.1124x+0.0261 | 1.000 |
| Mn | y=1.0965x+0.0833 | 0.999 |
| Co | y=4.7919x+0.0157 | 0.999 |
| Cu | y=3.9757x+1.2589 | 0.999 |
| Zn | y=0.6280x+1.8705 | 0.999 |
| As | y=0.4992x+0.0098 | 0.999 |
| Se | y=0.0244x+0.0141 | 0.999 |
| Sr | y=0.2200x+0.0115 | 0.999 |
| Cd | y=0.0330x+1.2971E-004 | 0.999 |
| Hg | y=0.0301x+7.1870E-005 | 0.999 |
| Tl | y=0.3397x+0.0043 | 0.999 |
| Pb | y=0.4520x+0.0239 | 0.999 |
